# Efficient Zinc Vanadate Homojunction with Cadmium Nanostructures for Photocatalytic Water Splitting and Hydrogen Evolution

**DOI:** 10.3390/nano14060492

**Published:** 2024-03-09

**Authors:** Imran Hasan, Adel El Marghany, Naaser A. Y. Abduh, Fahad A. Alharthi

**Affiliations:** Department of Chemistry, College of Science, King Saud University, Riyadh 11451, Saudi Arabia; amarghany@ksu.edu.sa (A.E.M.); 439106262@student.ksu.edu.sa (N.A.Y.A.)

**Keywords:** nanoparticles, cadmium nanostructures, H_2_ evolution, homojunction, photocatalysis

## Abstract

Construction of a homojunction is an effective strategy for effective charge transfer to suppress charge carrier recombination in augmented photocatalysis. The present work reveals the synthesis of homojunction formation through the reinforcement of Cd nanostructures into a solid lattice of zinc vanadate (Zn_3_V_2_O_8_, ZnV) using the hydrothermal method. The formation of a homojunction between cadmium vanadate (CdV, Cd_3_V_2_O_8_) and ZnV was confirmed by various spectroscopic and electron microscopic techniques such as Fourier-transform infrared (FTIR), X-ray diffraction (XRD), scanning electron microscopy (SEM) associated with energy-dispersive X-ray (EDX) mapping, transmission electron microscopy (TEM), X-ray photoelectron spectroscopy (XPS), and ultraviolet–visible spectrophotometry (UV–Vis). The synthesized material was explored for photocatalytic hydrogen (PC H_2_) production using the water splitting process under visible-light illumination. The spectroscopic and experimental results revealed that the formation of a CdV/ZnV homojunction significantly improved the transport of photogenerated charge carriers (electron–hole pairs) and thus resulted in enhanced H_2_ production efficiency (366.34 μmol g^−1^ h^−1^) as compared to pristine ZnV (229.09 μmol g^−1^ h^−1^) and CdV (274.91 μmol g^−1^ h^−1^) using methanol as a sacrificial reagent (SR) with water under visible-light illumination. The synergistic effect of Cd on ZnV NPs resulted in band gap reduction and broadened visible light absorption which was attributed to enhanced H_2_ production. The current study explains how a homojunction affects various features of important factors behind photocatalytic activity, which supports significant insights into the advancement of materials in the future.

## 1. Introduction

Rapid consumption of fossil fuels has been observed in the past few years, creating an energy crisis. The conventional energy resources being utilized at present are limited and lead to global warming issues [1,2,3]. Based on the above issues, the scientific community has been looking for an alternative and has found semiconductor photocatalysis based on the Honda–Fujishima effect [4]. Photocatalytic water splitting, an eco-friendly and an alternative method for fossil fuel consumption, reduces environmental pollution and the global warming issues, which are linked with energy production, by utilizing solar energy effectively [5,6,7]. Hydrogen (H_2_) production, an ecological and economical alternative, has attracted the attention of researchers. Various methods like the electrolysis of water, biomass gasification, fermentation, and steam reforming produce hydrogen and each has its own disadvantages [1,2,3]. The production of hydrogen by photocatalysis has been identified as one of the more effective and greener approaches for the proper usage of solar energy [3,8,9]. The products obtained by this method are environmentally friendly, carbon-free, and have a high calorific value. As solar energy consists of 40% visible light, in order to utilize it properly, semiconductors with a controlled electronic structure, a lattice structure, and high photocatalytic performance need to be developed [10].

Various semiconductor photocatalysts have been developed and applied in environmental remediation and energy catalysis including transition metal oxides (TiO_2_, ZnO, Fe_2_O_3_), sulfides (ZnS, CdS, MoS_2_), carbon nitrides (g-C_3_N_4_), oxysulfides (MoCuOS), oxynitrides (TaON), SrTiO_3_, and vanadates (BiVO_4_) [3,10,11,12,13,14]. Every material has its own merits and demerits. Among the above-mentioned materials, metal vanadates with general formulae M_3_V_2_O_8_, M = Zn, Cd, Ni, Cu, Co, etc., have garnered substantial interest due to their numerous properties like astonishing chemical constancy, low optical damage, higher catalytic activity, and easy availability [15,16,17,18,19,20]. They have been used in catalysis and energy-related applications [13,14,15,16,17,18,19,20,21,22,23,24]. In the case of Fe and Cu vanadates, the electronic structure comprises a valence band, a conduction band of O2p orbitals, and partially filled 3D orbitals, which extend the light absorption properties, thus resulting in efficient hydrogen production [19,20]. Zinc vanadate (Zn_3_V_2_O_8_) showcases remarkable optoelectronic properties, rendering it highly sought-after in various applications such as supercapacitors, batteries, hydrogen storage, catalysis, photocatalysis, and magnetic devices [16,17,22]. However, in the case of ZnV, the valence band has an O2p orbital and the conduction band has a V3d orbital. Because of the fully filled 3D orbitals of zinc, depicting a high recombination rate of photogenerated electron–hole pairs and the wide bandgap hampers its photocatalytic performance. There are some demerits; for example, its large band gap and its low surface area limit its photocatalytic efficiency. Interestingly, low-band-gap semiconductors effectively break water into hydrogen and oxygen, as most of the semiconductors with large band gaps are UV-active [16,17,18,19,20,21,22,23]. Recently, a few research groups have synthesized various vanadate materials and their composites and have employed them in environmental remediation and hydrogen production [25,26,27,28,29]. Expanding the utility of ZnV as a photocatalyst for H_2_ production necessitates augmenting its charge transfer pathways to mitigate the charge transfer recombination within the photocatalyst and thereby increasing the overall efficiency [28,29,30]. There are various research studies that have explored the strategies to enhance the photocatalytic efficacy of the materials by accelerating the segregation of charge carriers. These include techniques such as metal or non-metal doping [31], the integration of noble metals [32], morphology modulation [26], and composite fabrication [33]. The formation of junctions between two semiconductors has been recognized as a beneficial technique for enhancing the separation of charge carriers within the photocatalyst [34]. In general, photocatalytic semiconductor junctions fall in two primary categories: homojunction- and heterojunction-based semiconductor photocatalysts [35]. Homojunction semiconductor photocatalysts are created by integrating semiconductor interfaces with similar bandgap energies and chemical compositions which may differ in terms of crystal dimension or phase [36]. The interconnected semiconductor also exhibits diverse physical, optical, and electrical properties [37]. Various synthetic methods have been employed to prepare these homojunction photocatalysts including hydrothermal [29], electrodeposition [38], mechanical mixing, etc. [39,40]. Homojunction photocatalysts play a crucial role in the development of nanoscale photocatalytic devices with versatile applications across various fields [41]. Compared to photocatalysts synthesized using pristine materials, homojunction-based systems have shown significantly enhanced photocatalytic activities [36,37]. For example, they have greatly improved the efficiency of the processes such as H_2_ generation through water splitting and the conversion of greenhouse gases into valuable products and fuels [42]. Moreover, homojunction photocatalysts have made significant contributions to environmental remediation by efficiently degrading waterborne organic pollutants and removing harmful VOCs from the environment [43]. 

Herein, due to its excellent optical and chemical properties, cadmium vanadate (CdV) was chosen to form a homojunction with ZnV; we reported the formation of a homojunction of CdV/ZnV type through the hydrothermal method, and we explored its application towards photocatalytic H_2_ production under visible-light illumination. The material was characterized by structural, morphological, and spectroscopic techniques to understand how the Cd nanostructures on ZnV NPs improved its efficiency for enhanced H_2_ production. The effect of various reaction parameters such as type of sacrificial reagent, irradiation time, pH of the medium, and catalyst dose was observed on the photocatalytic H2 production efficiency of the synthesized material.

## 2. Materials and Methods

### 2.1. Reagents and Chemicals

Vanadium pentoxide (V_2_O_5_, ≥98%), Cadmium nitrate (Cd (NO_3_)_2_·4H_2_O, 98%), and Zinc nitrate (Zn (NO_3_)_2_·6H_2_O, reagent grade, 98%) were received from Sigma Aldrich (Burlington, MA, USA). Absolute ethanol (C_2_H_5_OH, 95%), sodium hydroxide (NaOH, ACS reagent, ≥97.0%, pellets), and Hydrogen peroxide (H_2_O_2_, 35%) were supplied by Loba Chemie, Mumbai, India. The chemicals received were of analytical grade and used without any further purification.

### 2.2. Synthesis of ZnV and CdV/ZnV

The hydrothermal synthesis method, as previously documented [16], was employed to produce both pristine ZnV and Cd–ZnV. Initially, 1.0 g of V_2_O_5_ was dispersed into 25 mL of deionized (DI) water, resulting in a bright yellow solution. Subsequently, 30% H_2_O_2_ was added dropwise, causing the solution to change to an orange hue. The mixture was left to magnetically stir for about 2 h to achieve homogeneous mixing. Following this, 0.5 g of Zn (NO_3_)_2_·6H_2_O and 0.2 g of Cd (NO_3_)_2_·4H_2_O were placed into 20 mL of DI water and added to the above mixture followed by an additional vigorous stirring for 45 min. Afterward, 0.5 M NaOH was introduced to the above mixture to maintain a pH value of 11–12. The resulting mixture was then transferred to a 100 mL Teflon-lined autoclave and heated at 170 °C for 15 h. Once the reaction completed, the autoclave was made to cool naturally at room temperature, and the precipitate was obtained through centrifugation. The material underwent several washes with DI water and absolute ethanol to eliminate the unreacted species, followed by drying at 90 °C for 4 h in a hot-air oven. Finally, the material was calcined at 550 °C for 4 h with a heating rate of 5 °C/min. Similarly, pristine ZnV was prepared using the same procedure, excluding the addition of cadmium.

### 2.3. Characterization Techniques

The synthesized samples ZnV and CdV/ZnV underwent comprehensive analysis using various analytical techniques to discern alterations in their physical and chemical properties pre- and post loading of the Cd nanostructures into pristine ZnV. X-ray diffraction (XRD) analysis was conducted using a Rigaku Ultima 1V X-ray diffractometer (Austin, TX, USA) while a Perkin Elmer Spectrum 2 FTIR spectrometer (Waltham, MA, USA) was employed for infrared spectroscopy. Morphological characterization was performed via scanning electron microscopy (SEM) using a JEOL GSM 6510 LV (Tokyo, Japan) and transmission electron microscopy (TEM) employing a JEM-2100 microscope (Tokyo, Japan). Optical properties were investigated using a Shimadzu UV-1900 double beam UV-vis spectrophotometer (Kyoto, Japan). Additionally, X-ray photoelectron microscopy (XPS) was employed to scrutinize the chemical state of constituent elements in the material, utilizing a PHI 5000 Versa Probe III instrument from Physical Electronics (Chanhassen, MN, USA).

### 2.4. Photocatalytic Hydrogen Production Experiments

A comparative investigation was conducted between ZnV, CdV, and CdV/ZnV to assess their efficiency in photocatalytic hydrogen (H_2_) production. The reaction took place in a photocatalytic reactor featuring a double-walled quartz reaction vessel, connected to a closed-gas circuit with a water jacket facilitating the maintenance of the reaction temperature at 10 °C. Before commencing the reaction, the reaction mixture underwent argon gas bubbling to eliminate oxygen and uphold anaerobic conditions. Subsequently, photocatalytic experiments were initiated by immersing 50 mg of synthesized nanomaterials in a solution containing 100 mL of a methanol–water mixture under illumination from a visible-light source (350 W Xenon lamp, 420 nm, 180 mW cm^−2^). The gas samples were obtained by using a 0.5 mL syringe from the headspace of the photoreactor and quantified using a multichannel analyzer (Emerson, St. Louis, MO, USA) furnished with a thermal conductivity detector. To quantify the H_2_ gas produced by photocatalytic reaction, an internal calibration method was employed. Photocatalytic tests were conducted for each sample ensuring a minimum of three repetitions for better accuracy and precision. The rates of H_2_ production were determined in μmol g^−1^ h^−1^ and the apparent quantum yield (AQY) was calculated by Equation (1):

(1) $$\mathrm{AQY}(\%)=\left(\frac{2\times {\mathrm{number}\;\mathrm{of}\;\mathrm{evolved}\;H}_{2}\;\mathrm{molecules}}{\mathrm{Number}\;\mathrm{of}\;\mathrm{incident}\;\mathrm{photons}}\right)\times 100$$

## 3. Results and Discussion

### 3.1. Material Characterization Studies

**Figure 1:**
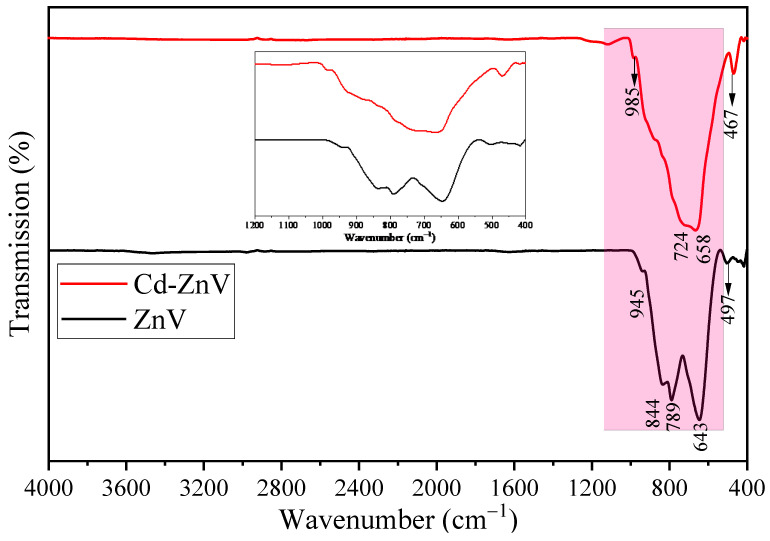
FTIR of ZnV (black line) and CdV/ZnV (red line).

The FTIR spectra of ZnV and CdV/ZnV are shown in Figure 1. The FTIR peaks at 945, 844, 789, 643, and 497 cm^−1^ show the characteristics of the ZnV sample. The peaks at 945, 844, and 643 cm^−1^ depict the V-O-V stretching, rocking, and vibration modes in VO_4_. The peak at 789 cm^−1^ results from the bonds shared by the atoms present in the corners of the VO_4_ in the tetrahedral structure, and the peak at 497 cm^−1^ is the typical stretching vibration of the V-O-Zn bond [22,26]. After modification of ZnV NPs with cadmium nanostructures, the characteristic peaks shown are similar to that of ZnV with a slight shift in the wavenumbers. The disappearance of peaks at 789 and 844 cm^−1^ takes place, and no other peaks from the reagents and cadmium-related impurities are observed, indicating the formation of a homojunction. As the samples are calcined above 500 °C, no characteristic peaks of O-H (adsorbed water) are observed. The inset image indicates that after reinforcement of Cd nanostructure in ZnV lattice, there appears a broad peak suggesting the formation of mix metal vanadate of type CdV/ZnV [25].

**Figure 2:**
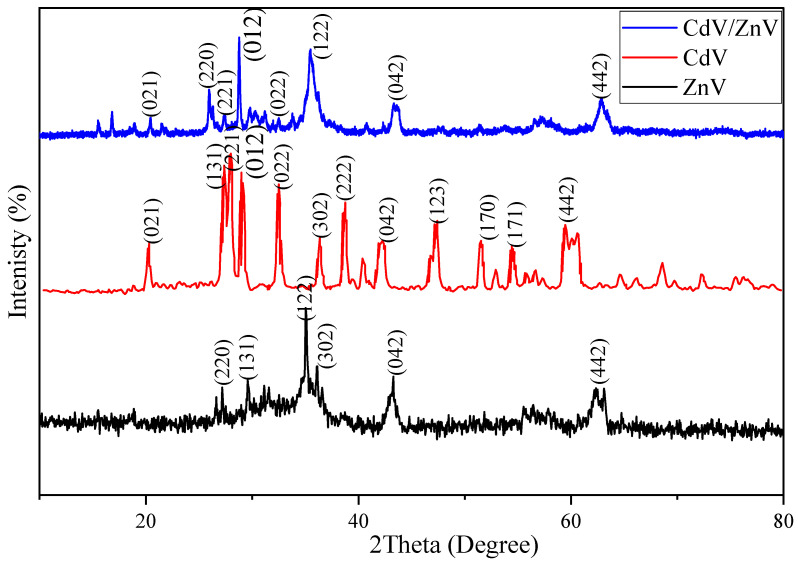
XRD of ZnV (black line), CdV (red line), and CdV/ZnV (blue line).

X-ray diffraction studies were carried out to examine the phase purity and crystal structure of ZnV, CdV, and CdV/ZnV NP samples calcined at 550 °C in the diffraction angle range of 10–80° (2*θ*) and are depicted in Figure 2. The XRD spectra of ZnV depict the characteristic peaks at 26.49, 29.59, 35.07, 36.20, 43.23, and 64.86, which correspond to Miller indices of (220), (131), (122), (302), (042), and (442) simulated with JCPDS card no. 34–0378. They reveal a characteristic typical spectrum of the orthorhombic ZnV phase and are consistent with earlier reports [26,27,28,29,30]. The XRD spectra of CdV exhibited the characteristic peaks at 2θ values of 20.29, 27.30, 27.95, 29.01, 32.52, 36.39, 38.73, 40.51, 42.52, 47.27, 51.59, 54.53, 59.61, 64.68, 68.59, and 72.43 corresponding to Miller indices of (021), (131), (221), (012), (022), (222), (042), (123), (170), (171), and (442) associated with a monoclinic structure simulated with JCPDS No. 78-0951. The XRD spectra of CdV/ZnV NPs reveal the characteristic peaks from both ZnV and CdV, which suggests that both Zn and Cd share the V_2_O_8_^6−^ lattice through the formation of a homojunction of type CdV/ZnV [40,44].

(2) $$D=\frac{0.9\lambda }{\beta \cos\theta }$$

The average crystallite size for CdV/ZnV was found to be 23 nm using the Scherer formula using Equation (2). As per the literature, narrow and sharp peaks depict good crystalline characteristics of the materials.

**Figure 3:**
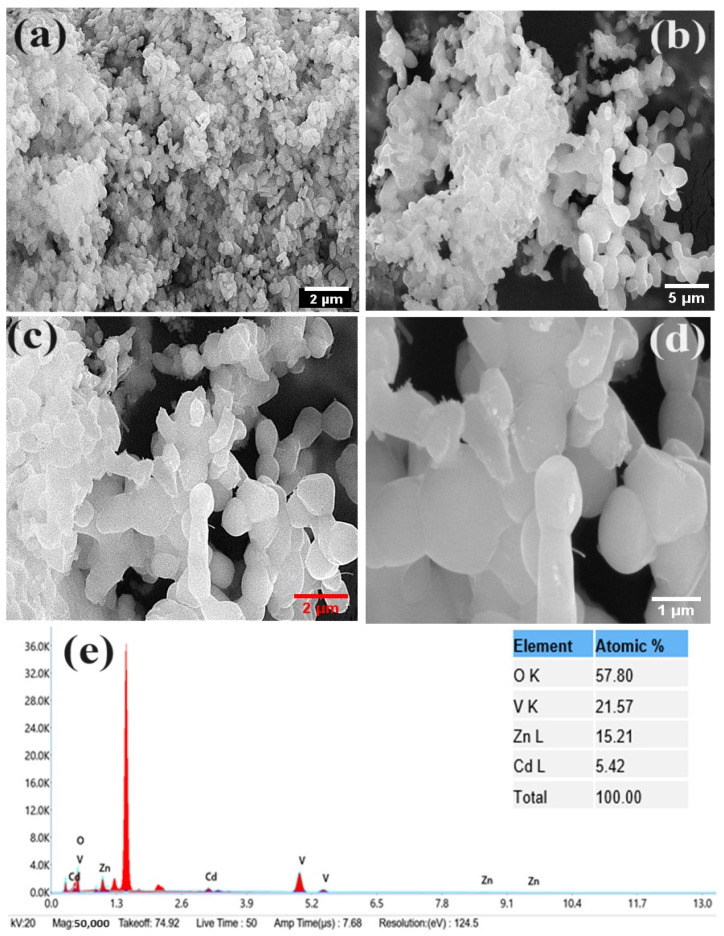
SEM image of (**a**) ZnV and (**b**–**d**) CdV/ZnV NPs, and (**e**) EDX spectrum of CdV/ZnV NPs.

**Figure 4:**
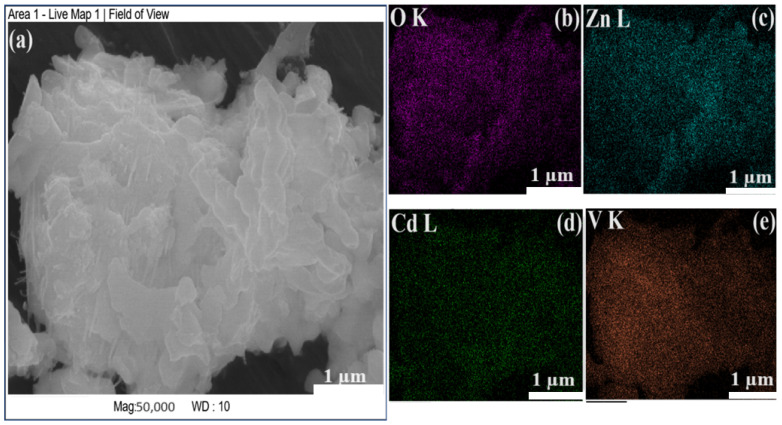
Selected area SEM image (**a**), and mapping image of O (**b**), Zn (**c**), Cd (**d**), and V (**e**) elements present in CdV/ZnV NPs.

Scanning electron microscopy (SEM) analysis of the as-synthesized materials was performed to evaluate the surface morphology and approximate particle size. Figure 3a depicts the dense array of small capsule-like morphologies of ZnV grown in all directions. Figure 3b–d represent the SEM image of CdV/ZnV NPs at different magnifications. Interestingly, homojunction formation between Cd and ZnV results in ellipsoid and spherical particle-like morphologies which are self-agglomerated and interconnected. The EDX analysis of CdV/ZnV NPs endorses the presence of corresponding elements in the material with atomic (%) as O (57.8), V (21.5), Zn (15.21), and Cd (5.42) respectively, which are shown in Figure 3e. No other elements were observed in the elemental mapping, depicting that the sample is free from impurities. XRD and SEM results support the formation of a homojunction between Cd and ZnV NPs. The selected area mapping image also confirms the presence of Zn, O, V, and Cd, ascribed to the uniform distribution of Cd nanostructures throughout the ZnV NPs and shown in Figure 4.

**Figure 5:**
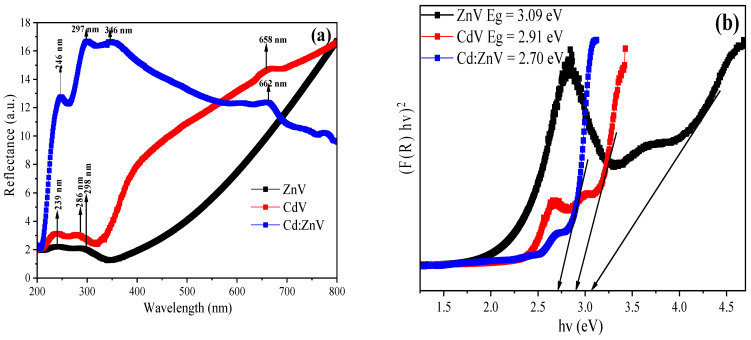
UV-vis reflectance spectra (**a**) and band gap plot (**b**) of ZnV and CdV/ZnV NPs.

The optical properties of the ZnV and CdV/ZnV nanostructures were studied by using UV-Vis diffuse reflectance spectroscopy (DRS) and the results are given in Figure 5a. The ZnV sample exhibited two reflective peaks in the range of 200–350 nm and one peak at 239 nm was observed due to the charge transfer from O to the central atom V in (V–O) bonds, while the second peak at 298 nm was ascribed to charge transfer from oxygen to zinc atoms in (Zn–O) bonds [30,45]. Similarly, the UV-DRS spectra of CdV exhibited two reflective peaks at 239 nm and 286 nm due to charge transfer between V–O and Cd–O bonds in the solid lattice [44]. One extra peak appears at 658 nm which is due to radiative charge transfer between Cd^2+^ and V_2_O_8_^6−^ in the Cd_3_V_2_O_8_ (CdV) [24,25]. The UV-DRS spectra of CdV/ZnV exhibited all the peaks from both ZnV and CdV with some shifted values suggesting the formation of a homojunction between ZnV and CdV. The appearance of a reflective peak at 658 nm suggested that the formed homojunction was visible-light-active. Further information about the optical properties of ZnV, CdV, and CdV/ZnV were elucidated by calculating the bandgaps of the materials using the Kubelka–Munk plot and is given by Equation (3) [46]:

(3) $$(F(R)hv)=A(hv-{\mathrm{Eg})}^{n}$$

where h is Planck’s constant, R is diffused reflectance, A is a constant, and *ν* is the frequency of radiations. In the equation, n corresponds to transition variations, and n is 2 for direct transitions and 1/2 for indirect transitions. A plot between F(R)hυ and hυ given in Figure 5b was used for the calculation of the energy bandgap of the synthesized nanomaterials. The bandgap values using the Kubelka–Munk equation were found to be 3.09 eV for ZnV, 2.91 eV for CdV, and 2.63 eV for CdV/ZnV, respectively. The obtained values suggested that homojunction formation between Cd and ZnV results in enhanced optical properties compared to pristine ZnV and CdV. Moreover, based on bandgap values, CdV is more optically active as compared to ZnV. The difference in band gap values upon homojunction formation is attributed to variation occupation of the lattice position of the dopant, type of electron transition, and crystalline size. These optical properties are directly related to the PC H_2_ production efficiency of the particular catalyst material. So, based on the results, the homojunction CdV/ZnV will exhibit a higher PC H_2_ production efficiency as compared to CdV, which in turn shows a higher efficiency than ZnV.

**Figure 6:**
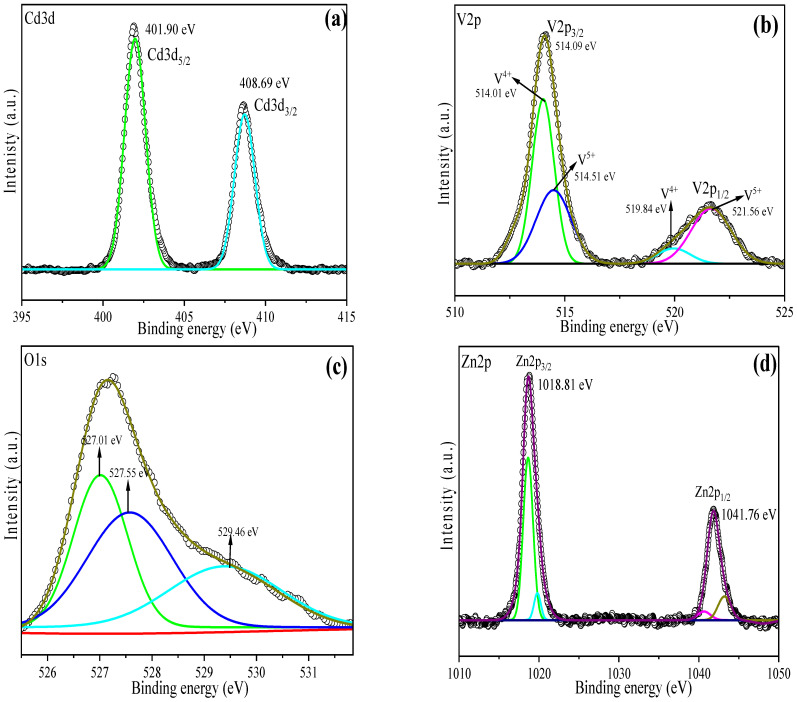
XPS deconvoluted fitted spectrum of Cd3d (**a**), V2p (**b**), O1s (**c**), and Zn2p (**d**) of CdV/ZnV NPs.

X-ray photoelectron spectroscopy (XPS) analysis was carried out to observe the surface electronic and chemical states of the elements. Figure 6a illustrates the high-resolution spectra of the Cd 3d core level. It can be deconvoluted into two peaks 401.9 and 408.69 eV, ascribed to the Cd 3d_5/2_ and Cd 3d_3/2_ spin states, respectively, which are the characteristics of the Cd^2+^ oxidation state [44]. Figure 6b displays the high-resolution spectra of the V 2p core level and can be deconvoluted into two peaks 514 and 521.09 eV attributed to V2p_3/2_ and V2p_1/2_, respectively, which are the characteristics of V^4+^ and V^5+^ oxidation states [28]. Figure 6c displays the high-resolution spectra of the O 1s core level and is deconvoluted into three peaks 527.01, 527.55, and 529.46 eV associated with V-O and Zn-O/Cd-O, along with a large number of defect sites [17]. Figure 4d shows the high-resolution spectra of the Zn 2p core level. It can be deconvoluted into two peaks 1018.81 and 1041.76 eV ascribed to Zn 2p_3/2_ and Zn 2p_1/2_, respectively, which are the characteristics of the Zn^2+^ oxidation state [18]. The binding energies of Zn, V, and O shifted to the lower side, ascribed to the formation of a homojunction with Cd nanostructures. The elemental composition in the sample depicts the atomic concentration of corresponding elements in the sample, Cd (7.06%), Zn (14.72%), V (20.65%), and O (57.57%), and are shown in Table 1.

**Figure 7:**
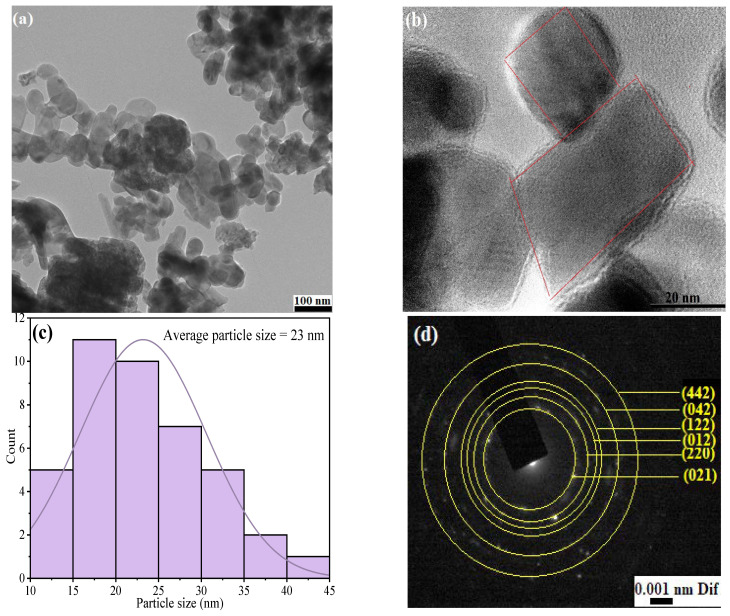
(**a**,**b**) TEM images of CdV/ZnV NPs at variable magnification values, (**c**) Gaussian distribution profile for particle size, (**d**) selected area electron diffraction (SAED).

TEM analysis was performed to evaluate the shape and orientation of the nanoparticles. Figure 7a,b represent the TEM image of CdV/ZnV NPs, which shows the random orientation of nanoparticles which, upon further magnification, exhibit two types of particle shapes: one is distorted monoclinic due to CdV and the other is distorted orthorhombic due to ZnV. So, TEM analysis also supported the formation of the homojunction between Cd and ZnV. Figure 7c shows the Gaussian distribution profile for calculating the average particle size of the nanoparticles. The average diameter of the nanoparticles was found to be 23 nm using the Gaussian distribution graph, which is consistent with Scherer’s calculated crystallite size. Figure 7d shows the SAED pattern of CdV/ZnV, which reveals the Miller indices of (042), (331), (200), (101), (220), and (301) and are consistent with XRD data, which indicates the presence of Cd nanostructures on ZnV.

**Figure 8:**
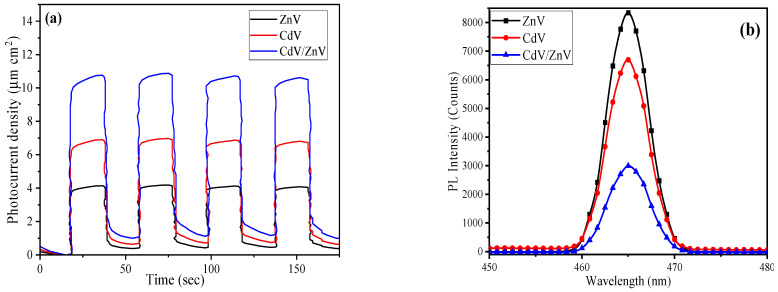
(**a**) Photocurrent response and (**b**) photoluminescence (PL) spectra for ZnV, CdV, and CdV/ZnV.

Further, to ensure the electron–hole charge separation, charge transport, and electrochemical stability of the synthesized material, transient photocurrent response analysis was considered. Figure 8a represents the photocurrent responses of ZnV, CdV, and CdV/ZnV with on/off cycles under visible-light irradiation. It can be seen that CdV/ZnV NPs exhibit a higher photocurrent density as compared to ZnV and CdV, which suggests that homojunction formation between Cd and ZnV results in an enhanced charge separation efficiency between electrons and holes, which in turn reflects the higher PC H_2_ production efficiency.

The photoluminescence spectra of ZnV, CdV, and CdV/ZnV were analyzed to observe the dynamics of electron–hole pair recombination and the results are given in Figure 8b. All the photocatalysts exhibit a strong emission peak between 460 and 470 nm in which ZnV possesses the highest emission peak, suggesting the highest electron–hole recombination rate and in turn reflecting the lowest PC H_2_ production efficiency. The CdV in comparison to ZnV possesses a lower PL intensity and thus exhibits a higher PC H_2_ production activity. The homojunction CdV/ZnV exhibited the lowest PL intensity, indicating the highest electron–hole pair separation efficiency and thus showing the highest PC H_2_ production activity.

### 3.2. Hydrogen Production Experiments

#### 3.2.1. Selection of Sacrificial Reagent

**Figure 9:**
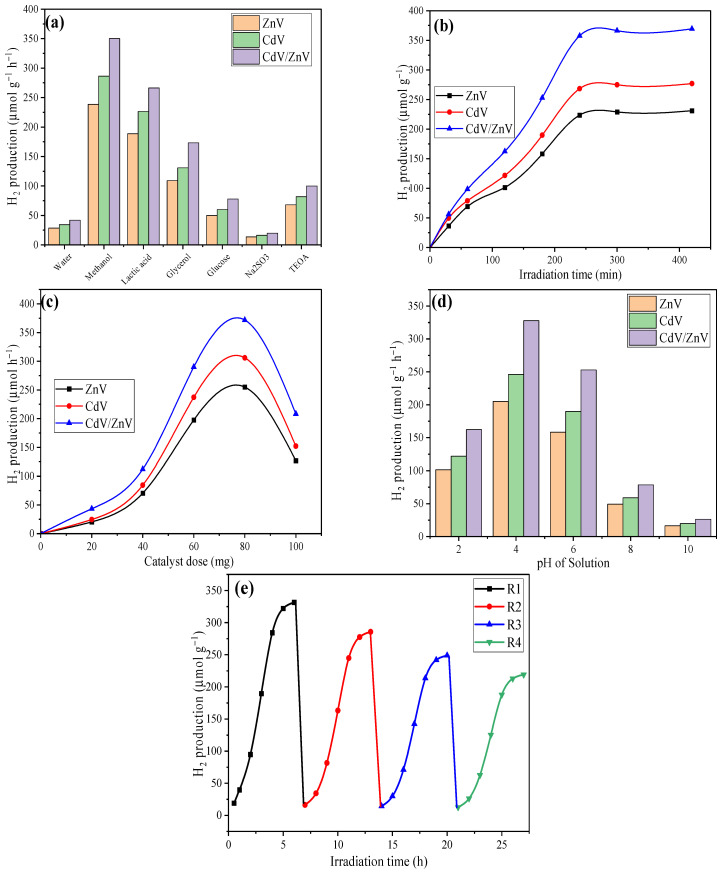
Effect of (**a**) various sacrificial reagents, (**b**) irradiation time (min), (**c**) catalyst dose, (**d**) pH of the solution (methanol + water), and (**e**) reusability experiments for ZnV and CdV/ZnV for PC H_2_ production reaction.

The effect of a sacrificial reagent (SR) in the hydrogen production reaction in the photocatalytic water-splitting process was studied with different SRs such as pure water, methanol, lactic acid, glycerol, glucose, Na_2_SO_3_, and TEOA in a photochemical reactor under UV-light illumination. The experiment was performed by dispersing 50 mg of ZnV, CdV, and CdV/ZnV into 100 mL (80 mL water + 20 mL SR) under visible-light illumination for 5 h. The rate of hydrogen production over various SRs follows the order methanol > lactic acid > glycerol > TEOA > glucose > water > Na_2_SO_3_ and the results are given in Figure 9a. Out of various SRs, methanol showed the highest H_2_ production of 238.66, 289.39, and 350.05 µmol g^−1^ h^−1^ over ZnV, CdV, and CdV/ZnV, and Na_2_SO_3_ exhibited the least hydrogen production of 13.68, 16.42, and 20.03 µmol g^−1^ h^−1^ over ZnV, CdV, and CdV/ZnV NPs, respectively. From the above results, methanol was chosen as a sacrificial reagent for the remaining PC experiments for optimizing various reaction parameters like illumination time, pH of solution, and amount of catalyst.

#### 3.2.2. Effect of Various Reaction Parameters

Subsequently, the effect of irradiation time was also studied, from 30 min to 300 min on the PC H_2_ production efficiency of the synthesized material. The rate of H_2_ production exhibited an increasing trend with an increase in irradiation time, revealing the values of 229.09 µmol h^−1^ for ZnV, 274.91 µmol h^−1^ for CdV, and 366.34 µmol h^−1^ for CdV/ZnV in a time interval of 300 min or 5 h. Further increasing the illumination time beyond 300 min displayed no appreciable change in H_2_ production efficiency. So, 300 min was taken as the optimum time for maximum H_2_ production activity. The results obtained are given in Figure 9b. As the irradiation time increases, owing to photoabsorption, the material reaction results in the generation of a higher number of electron–hole pairs, which results in higher H_2_ production efficiency [9].

PC experiments were carried out by altering the catalyst amount from 20 mg to 100 mg under visible-light illumination by taking 20 mL of methanol and an 80 mL water solution for a time interval of 5 h, and the results are shown in Figure 9c. With the increase in catalyst dose from 20 to 80 mg, the rate of H_2_ production increased from 43.33 to 371.68 µmol h^−1^; upon the further increment in the catalyst to 100 mg, a sharp decrease in the value to 208.23 µmol h^−1^ over CdV/ZnV was observed. The same trend was observed over ZnV and CdV catalysts, showing a maximum H_2_ production of 255 and 306 µmol h^−1^ with the final value of 126.82 and 152.19 µmol h^−1^, respectively. The increasing and decreasing trend can be attributed to the more active sites which can absorb the radiation and generate more electron–hole pairs for water splitting. Nonetheless, upon reaching a maximum limit, the solution becomes turbid due to the accumulation of nanoparticles which scatter and reflect the light radiation, reaching the surface of the catalyst and resulting in hampered H_2_ production [12].

Consequently, the effect of pH of the solution was studied on ZnV, CdV, and CdV/ZnV catalysts and is demonstrated in Figure 9d. When the pH of the solution is highly acidic (pH = 2, highly acidic), the rate of H_2_ production is 101.45, 121.45, and 162.23 µmol g^−1^ h^−1^; upon the further decrease in acidity (pH = 4, moderately acidic), the H_2_ production enhanced and exhibited a value of 205.09, 246.11, and 327.95 µmol g^−1^ h^−1^; and upon further dilution (pH = 6, weakly acidic), the H_2_ production decreased and was found to be 158.18, 189.82, and 252.94 µmol g^−1^ h^−1^ on ZnV, CdV, and CdV/ZnV, respectively. However, increasing the rate of H_2_ production further decreased them to 49.09, 58.91, and 78.50 at pH 8 and 16.39, 19.63, and 26.17 µmol g^−1^ h^−1^ at pH 10. The results revealed that highly acidic or highly basic conditions suppressed the H_2_ production due to the deviation from the positive surface of the catalyst and the lower availability of H^+^ ions [19]. The moderately acidic conditions are favorable for enhanced hydrogen production. Further, experiments were performed to check the stability of the synthesized nanomaterial towards photocatalytic H_2_ production in a cyclic mode. After every experiment, the catalyst was centrifuged, washed with DI water, dried in the oven, and used for the next experiments. The stability experiments were carried out for four cycles, and the results are given in Figure 9e. It can be seen that with each cycle of reuse, there is a very small magnitude of decrease in photocatalytic H_2_ production efficiency of the material, which is due to photocorrosion from Cd nanostructures. This phenomenon suggests that the material has optimum stability towards photocatalytic water splitting and H_2_ production. The apparent quantum yield (%) using Equation (1) was calculated as 1.63% for ZnV, 1.76% for CdV, and 2.11% for CdV/ZnV. The results obtained suggested that the homojunction formation of ZnV with the Cd nanostructure resulted in enhanced photocatalytic H_2_ production efficiency and apparent quantum yield.

### 3.3. Mechanism of Reaction

**Scheme 1:**
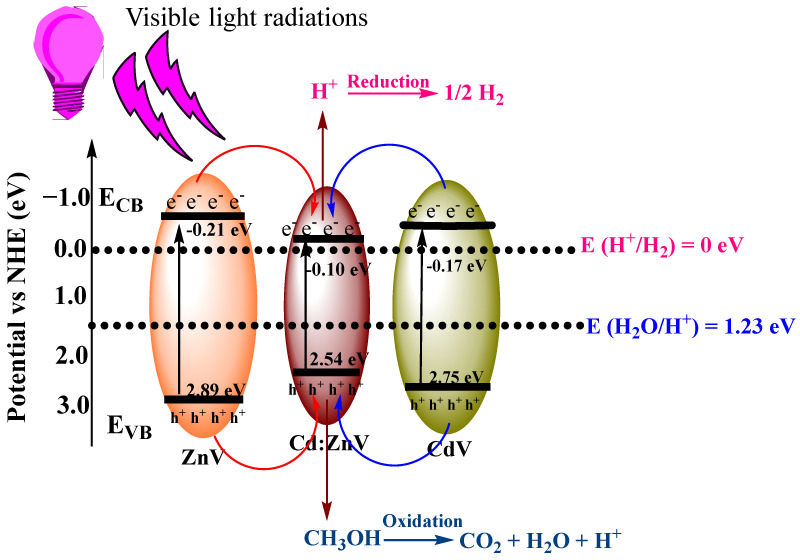
Schematic representation of H_2_ gas production by CdV/ZnV NPs using methanol as sacrificial reagent.

The overall process of water splitting involves two sequential half reactions: reduction and then oxidation. To facilitate the water splitting reaction, the photocatalyst conduction band potential should be more negative than the reduction potential of hydrogen, enabling the reduction half-reaction. Similarly, for successful oxidation, the valence band potential of the catalyst should be more positive than the oxidation potential of water, which is 1.23 eV [47]. However, oxygen generation may not always occur during water-splitting reactions, due to the presence of sacrificial reagents which serve to consume the hole h^+^ on the surface of the catalysts and thus influence the overall efficiency of the process [48]. Scheme 1 depicts the possible mechanism for the photocatalytic water splitting and methanol oxidation reaction over ZnV, CdV, and CdV/ZnV homojunctions under visible-light illumination. Optical studies reveal that the homojunction formation of ZnV with the Cd nanostructure results in a reduction in energy bandgap from 3.09 eV to 2.63 eV with extended light absorption properties. The modification also reflects on the experimental studies by depicting the higher PC H_2_ production efficiency of CdV/ZnV over ZnV. Mulliken’s electronegativity theory was used to calculate the potentials of VB and CB of the synthesized material by using the following equations:

(4) $$E_{\mathrm{VB}}=\chi -E^{\left(e\right)}+0.5\times E_{g}$$

(5) $$E_{\mathrm{CB}}=E_{\mathrm{VB}}-E_{g}$$

where χ is Mulliken’s electronegativity, E^(e)^ is the energy of the free electron (4.5 eV), and E_g_ is the energy bandgap of material obtained through Tauc’s plot. Using Equations (4) and (5), the valence band (VB) and conduction band (CB) potentials were found to be 2.89 eV and −0.21 eV for ZnV, 2.75 eV and −0.17 eV for CdV, and 2.54 eV and −0.10 eV for CdV/ZnV NPs, respectively. It can be seen that the CB of CdV/ZnV is lower than that of both ZnV and CdV and more negative than the reduction potential of hydrogen (H^+^/H_2_ = 0 eV). When the material is illuminated by visible-light radiation within the vicinity of the methanol–water solution, the generation of photogenerated charge carriers occurs, i.e., electron–hole pairs on the surface of the photocatalysts ZnV, CdV, and CdV/ZnV. Since the CB potential of ZnV and CdV is lower than that of the CdV/ZnV homojunction, the photogenerated electrons will transfer to the CB of CdV/ZnV and, similarly, the holes created will also migrate towards the VB of CdV/ZnV. The holes in the VB of CdV/ZnV react with water and oxidize it to form proton and hydroxyl radicals. These hydroxyl radicals react with methanol to form CO_2_ and water. Thus, the presence of SR in the photocatalytic process increases the H_2_ production efficiency by suppressing the charge carrier recombination. The electrons in the conduction band react with protons generated in the oxidation process and reduce them to hydrogen gas. The plausible mechanism for PC H_2_ production over CdV/ZnV can be represented by Equations (6)–(9).

(6) $$\mathrm{CdV}/\mathrm{ZnV}\overset{\mathrm{hv}}{\to }{\mathrm{CdV}/\mathrm{ZnV}}^{*}{(h}_{\mathrm{VB}}^{+}+e_{\mathrm{CB}}^{-})$$

(7) CdV/ZnV*(hVB+)+H2O→H++O•H

(8) $${\mathrm{CdV}/\mathrm{ZnV}}^{*}{(e}_{\mathrm{CB}}^{-}{)+H}^{+}\to {1/2H}_{2}$$

(9) O•H+Methanol→H2O+CO2

### 3.4. Comparison with Literature

The outcomes of the present study were compared with the reported data in the literature and data are given in Table 2. It can be seen that the material synthesized in the present study exhibited very high PC H_2_ production efficiency as compared to other photocatalysts involving Zn-based nanomaterials.

## 4. Conclusions

The current article reports the hydrothermal synthesis of cadmium nanostructures on zinc vanadate. FTIR studies and structural studies depicted the formation of an orthorhombic phase structure along with cadmium nanostructures with a uniform average crystallite size of 23 nm. EDX and XPS studies depicted the uniform distribution of Cd on ZnV, the composition, and the oxidation states of the respective elements. Diffuse reflectance spectroscopic studies suggested the broadened visible-light absorption of the materials with a band gap of 2.63 eV. The photocurrent response and PL spectra also suggested that the synthesized homojunction CdV/ZnV materials resulted in high electron–hole separation efficiency as compared to pristine CdV and ZnV. The synthesized materials ZnV, CdV, and CdV/ZnV exhibited a hydrogen production rate of 229.09, 274.91, and 366.34 µmol g^−1^ h^−1^ at pH 4 for 5 h of visible-light illumination in the presence of methanol as a sacrificial reagent. The material was found to be stable and reusable for up to four cycles. The formation of a homojunction between ZnV and Cd leads to high hydrogen production due to the suppression of recombination, thus increasing the lifetime of charge carriers.

## Figures and Tables

**Table 1:** Atomic concentration table.

**O1s**	**V2p3**	**Zn2p3**	**Cd3d5**	RSF Corrected
57.57	20.65	14.72	7.06	

**Table 2:** Comparison of outcomes of present study with literature.

Photocatalyst	Light Used	SR	H_2_ Production (µmol g^−1^ h^−1^)	Reference
ZnO@C/g-C3N4	simulated sunlight	TEOA	336.08	[49]
Ag/ZnO	mercury xenon lamp	Ethanol	12.00	[50]
ZnS@CuS	Visible		56.47	[51]
ZnO/ZnS-3h-NiS	Visible	Ethanol	26.00	[52]
WO3-x/ZnIn2S4	NIR	TEOA	14.05	[53]
ZPCN	Visible	Methanol	90.00	[54]
CoMoO4/GDY	Visible	TEOA	121.00	[55]
CdV/ZnV	Visible	Methanol	366.34	Present study

## Data Availability

Data are contained within the article.
